# A systems biology approach for the identification of target genes for the improvement of itaconic acid production in *Aspergillus species*

**DOI:** 10.1186/1756-0500-6-505

**Published:** 2013-12-04

**Authors:** An Li, Martien Caspers, Peter Punt

**Affiliations:** 1 TNO Microbiology and Systems biology, PO Box 360, Zeist, AJ 3700, The Netherlands

**Keywords:** Itaconic acid, Transcriptomics, *Aspergillus terreus*, *Aspergillus*

## Abstract

**Background:**

In this paper, a clone based transcriptome analysis towards the identification of genes related to itaconic acid production in *Aspergillus terreus* was carried out as an extension of a previously published a clone-based transcriptome analysis from a set of batch fermentation experiments. Also a publically available *A. niger* transcriptome dataset from cultures similar to those of the *A. terreus* data set was analyzed to evaluate the specificity of the approach followed for *A. terreus*.

**Results:**

Besides the itaconic acid gene cluster (*cis*-aconitate decarboxylase, mitochondrial tri-carboxylic acid transporter and major facilitator superfamily transporter) discovered previously, additional genes of interest were identified in the *A. terreus* transcriptome data correlating to itaconic acid production, including 6 genes encoding enzymes in glycolysis and the pentose phosphate pathway, 4 genes functioning in vitamins synthesis, and a gene encoding a copper transporter. Only three of the 83 low pH specific genes identified from the *A. niger* dataset corresponded to high itaconic acid / low pH expressed genes identified from the *A. terreus* data set. However, in all three cases, the regulation of pH dependent gene expression was completely different between the two species.

**Conclusions:**

An extended clone based transcriptome analysis using a clone based transcription array to identify genes correlating with itaconic acid production revealed novel genes both in the central metabolism and in other more secondary pathways such as vitamin biosynthesis and Cu^2+^ transport, providing targets for further metabolic and process engineering to optimize itaconic acid production.

## Background

Itaconic acid as a building block chemical is used in industry [1-4]. Currently, the most commonly used production host for this compound is *Aspergillus terreus*. However, its market price is relatively high which restricts its broader industrial application [2]. Therefore, a new production host – *Aspergillus niger* was introduced for producing itaconic acid towards more economical levels [5]. *Aspergillus niger,* is well known for its high capacity on citric acid (precursor of itaconic acid) production and a high efficiency for converting its carbon source into organic acids [6]. Moreover, *A. niger* is capable of consuming different carbon sources (glucose, xylose, glycerol etc.) and is quite robust in various industrial environments [5-9]. To produce itaconic acid in an economical feasible way, either *A. terreus* or *A. niger* still requires extensive metabolic reprogramming to improve conversion of the supplied carbon source or enhanced flux from citric acid towards itaconic acid. Engineering of these complex traits will be facilitated by an in-depth understanding of the metabolism of the two *Aspergillus* systems, considering metabolic and genetic networks as a whole. Such knowledge is being generated by taking an integrated systems biology approach using current transcriptomics tools. Based on the outcome of this study and using the available genetic engineering tools, the production pathway of itaconic acid from *A. terreus* can be constructed in the new production host *A. niger*.

As discussed in our previous study [10], the biological pathway of itaconic acid is rather complicated due to its two subcellular locations. The glycolysis part from glucose to pyruvate occurs in the cytosol, and then the conversion from pyruvate to *cis*-aconitate takes place in the mitochondria. Since the next step, from *cis*-aconitate to itaconic acid catalyzed by *cis*-aconitate decarboxylase (CAD) is again occurring in the cytosol, transport of *cis*-aconitate from the mitochondria to the cytosol is required. Then, the produced itaconic acid has to be transported out of the cell. In this whole pathway, the three genes encoded by the highly up-regulated gene cluster are assumed to be involved. The discovery of the *cadA* gene cluster in *A. terreus* opened the genetic modification targets for itaconic acid production in *A. niger*.

In this study, identifying genes relevant to itaconic acid and possibly other primary metabolism products, was used to study the network governing organic acid production in *A. terreus* and in *A. niger*. Transcriptome data was used to identify genes in *A. terreus* related to high yield itaconic acid production conditions. These were identified using clone based microarrays from a set of batch fermentation experiments [11]. As described in this study, itaconic acid production could be influenced by environmental factors such as nutrient, pH, etc. [1-4]. Therefore, we assumed that the expression level of genes highly related to itaconic acid production would be influenced by these environmental factors. The batch cultivations were carried out with various carbon sources, different levels of trace elements (e.g. Mn^2+^), pH and dissolved oxygen supplies, which resulted in different levels of itaconic acid production. Among them, pH had a strong influence on the production level ([11], Additional file 1: Table S1). Based on these results, for studying the influence of environmental factors on organic acid production in general, we also analyzed an available data set [12] consisting of the expression profiles from *A. niger* cultures performed under three different pH conditions (2.5, 4.5 and 6) with glucose as a carbon source. As the cultivation conditions were quite similar for itaconic- production in *A. terreus* and citric acid production in *A. niger*, both had culture pH around 2 and glucose supplied as a carbon source. Comparing the two transcriptomics data sets would possibly allow the identification and discrimination of genes specific for itaconic acid production, versus those specific for only culture pH. These genes might provide new targets for further strain development for our research on producing itaconic acid in *Aspergillus*[13].

## Methods

### Transcriptomics data analysis

#### Clone-based transcriptome data (*A. terreus*)

As described in our previous research [11] using a cDNA clone based approach for target gene identification, cDNA clones were selected based on their differential expression ratios on both titer and productivity. In the previous study the minimum ratio used for gene identification was set to 10, resulting in the identification of the itaconic acid gene cluster [11]. Here, we expanded this selection to genes showing a differential expression ratio ≥ 2 or ≤ 0.5 in either titer or productivity. After this expanded selection the inserts of the identified clones were sequenced (BaseClear) and identified by BLAST comparison against the NCBI database.

#### Andersen’s data (*A. niger*)

To select genes specific for low pH conditions and thus presumably for organic acid production, a publically available data set from pH 2.5 cultures was compared to a data set from pH 4.5 cultures [12]. For this selection we used a relatively stringent threshold ratio of 4 (2Log = 2) between expression values obtained at pH 2.5 and pH 4.5.

## Results

### An extended *A. terreus* transcriptome analysis

**Figure 1:**
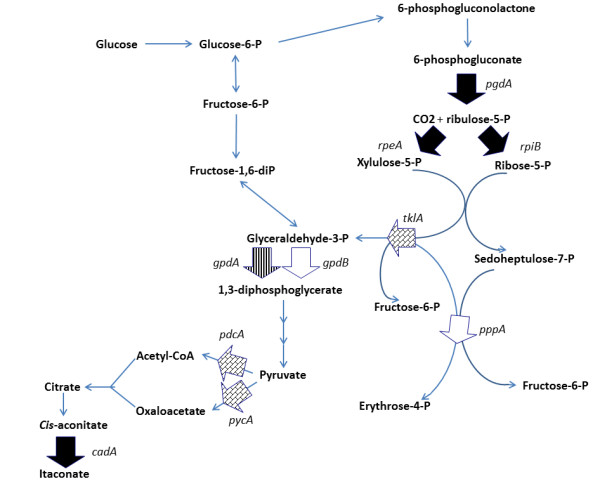
**Schematic representation of Glycolysis and pentose phosphate pathway*.*** Broad arrows indicate the enzymes (indicated by the names of the corresponding genes) highly related to itaconic acid production as identified from the *A. terreus* clone based transcriptome data analysis (see Additional file 1: Table S1). Group I (black arrows) *pgdA* (6-phosphogluconate dehydrogenase), *rpeA* (ribulose-phosphate 3-epimerase), *rpiB* (ribose 5-phosphate isomerase), *cadA* (*cis*-aconitate decarboxylase), group II (vertically striped arrow) *gpdA* (glyceraldehyde-3-phosphate dehydrogenase)*,* group III (white arrows) *gpdB* (glyceraldehyde-3-phosphate dehydrogenase), *pppA* (transaldolase). The vitamins dependent *tklA* (transketolase), *pycA* (pyruvate carboxylase) and *pdcA* (pyruvate decarboxylase) (thus related to the upregulated vitamin biosynthetic genes in group II and IV) were indicated with the blocked filled arrows.

As already presented in our previous study, initial transcriptomics analysis of *A. terreus* using a clone-based microarray discovered the *cadA* gene cluster, consisted of *cadA* (ATEG_09971), 1 mitochondrial tri-carboxylic acid transporter (ATEG_09970) and 1 major facilitator superfamily transporter (ATEG_09972) [11,13]. Further differential analysis on itaconic acid titer and productivity identified 165 clones belonging to 33 genes (Additional file 1: Table S1). In Additional file 1: Table S1, the maximal expression value of each gene was listed (MaxExpr/Gene) and the relative expressions of the same gene from different cultivation conditions/time were scored based on its maximum expression (MaxExpr is set to be 1). The relative expressions were sorted in relation to itaconic acid production levels. Besides the previously identified *cadA* cluster genes, several other genes were identified: 4 genes encoding enzymes in the Pentose Phosphate (PP) pathway, 2 glycolysis pathway genes (*gpdA*, *gpdB,*[14]), (Figure 1), 4 vitamin biosynthesis genes, 1 gene (ATEG_09368) encoding a second transporter belonging to the Major Facilitator Superfamily (MFS) and 1 gene (ATEG_06726) encoding a pH responsive plasma transmembrane 1,3-glucanosyltransferase (description from AspGD [15]). In Additional file 1: Table S1, these 33 genes were clustered based on their relative expression profile in a range of conditions analysed in Li et al. [11] using Pearson Correlation [16]. Clustering resulted in four groups (I, II, III and IV). The 3 Itaconic acid cluster genes were present in group I where genes with high expression positively correlated with only the highest itaconic acid production condition. Group II genes also have relatively high expression in highest itaconic acid production conditions but also in lower itaconic acid level production conditions. Group III consists of genes negatively correlating with itaconic production, The 2 remaining genes (group IV) show an expression profile clearly unrelated to any other three groups showing particularly low expression at pH 4.5 where hardly any itaconic acid was produced. In group I, 3 PP pathway genes (encoding 6-phosphogluconate dehydrogenase, ribulose-phosphate 3-epimerase and fructose-bisphosphate aldolase) were present showing a similar profile as the three genes of the itaconic acid *cadA* gene cluster. In group II, glyceraldehyde3-phosphate dehydrogenase (*gpdA*) which might be considered as an upstream protein from the itaconic acid pathway was present. Furthermore, a copper transporter gene (ATEG_08509) also clustered in this group. In groups I and II, also several genes encoding uncharacterized proteins with predicted plasma membrane or extracellular localization were present.

Interestingly in Group III, a fourth PP pathway gene encoding transaldolase and an alternative glyceraldehyde 3-phosphate dehydrogenase *gpdB* were present.

In the various groups, 4 genes involved in vitamins biosynthesis were present. The plasma membrane pyridoxine biosynthesis protein PDX1 (ATEG_08317) in group I represents a step in the biosynthesis pathway of pyridoxine (vitamin B6). In group II, 2 thiamin (vitamin B1) biosynthesis pathway genes (ATEG_05818, ATEG_09213) were present. The biotin (vitamin H) synthase gene (ATEG_07101) was present in group IV.

### Analysis on a pH based *A. niger* transcriptome data set

As observed from the results of the *A. terreus* itaconic acid fermentations [11], pH strongly influenced the itaconic acid production level and related gene expression. In an attempt to analyze whether any of the genes identified from the *A. terreus* arrays are actually pH and not itaconic acid related, microarray data from Andersen’s *A. niger* cultivation under two pH levels were compared with our results from the *A. terreus* clone-based arrays. As a first step, we analyzed expression levels of the genes involved in the pathway leading to itaconic acid precursors from the TCA cycle, including the genes encoding citrate synthase, aconitase and cadA/prpD like proteins. This analysis showed that their expression levels were nearly the same in all three pH conditions (results not shown).

In a second approach we analyzed *A. niger* genes which were expressed in a pH dependent manner and whether these corresponded to any of the genes listed in Additional file 2. Our previous study already indicated that itaconic acid production was influenced by pH where the production level is maximal around pH 2.3 and minimal at pH 4.5, so we focused on the genes of which expression levels differed at pH 2.5 and 4.5. In total, 83 *A. niger* genes were selected based our selection criteria (Andersen et al., [12]; results not shown). Using BLAST analysis against *A. terreus* accessions present at NCBI bidirectional best hits to *A. terreus* were identified for these *A. niger* genes. Only three of these genes corresponded to the genes identified from the *A. terreus* arrays as described in Additional file 1: Table S1. Among them, two genes An09g00630 (CipC protein) and An05g02250 (GPI-anchored protein) were up-regulated in pH 2.5 in comparison to pH 4.5 whereas gene An02g11360 (endo alpha-1,4 polygalactosaminidase) was down-regulated in pH 2.5, whereas the same genes in *A. terreus* showed the opposite expression profile.

## Discussion

Using a clone based transcriptome analysis, we aimed to obtain a better understanding of the biological production pathway of itaconic acid in *A. terreus*, and part of the corresponding pathway present in *A. niger*.

In this paper more detailed analysis of the transcriptome data was carried out to identify additional genes related to itaconic acid production. The identification of three up-regulated PP pathway genes in the same group as the itaconic acid pathway genes (group I; Additional file 1: Table S1) suggested that part of the PP pathway enzymes (Figure 1) positively influence the carbon flux into the itaconic acid production pathway. Also the fact that thiamin biosynthetic genes positively correlated with itaconic acid biosynthesis corresponds with the fact that the PP pathway protein transketolase (*tklA* Figure 1) is thiamin dependent. Interestingly, the PP Pathway was found to be not involved in the biosynthesis of itaconic acid from research carried out previously [1]. In our case, the identified PP pathway genes might also suggest a side metabolic route from glucose to glyceraldehyde-3-phosphate via a part of the PP pathway (Figure 1). Furthermore, another study based on metabolic modeling also suggested a role of the PP pathway on itaconic acid production [17]. Therefore, these identified PP pathway genes are highly interesting for future genetic pathway modification for improving itaconic acid production level. In group II the identification of the *gpdA* gene encoding glyceraldehyde-3-phosphate dehydrogenase suggested a role of glycolysis in itaconic acid production possibly influencing the synthesis of citrate (the precursor of itaconic acid). Glyceraldehyde-3-phosphate dehydrogenase might also influence the activity of the PP pathway, since its substrate glyceraldehyde-3-phosphate is derived from this pathway (Figure 1). However, although *gpdA* is up-regulated under itaconic acid producing conditions, over-expression of the *A. niger gpdA* gene did not improve itaconic acid production in an *A. niger* itaconic acid producing strain [5]. Surprisingly an alternative glyceraldehyde-3-phosphate dehydrogenase (*gpdB*) was identified in Group III. Interestingly in group III, also a down-regulated gene encoding the PP pathway protein transaldolase (Figure 1) was identified. Transaldolase converts glyceraldehyde-3-phosphate originating from the glycolysis pathway, and reducing its expression may increase the flux towards itaconic acid production. With respect to the role of glycolysis the identification of thiamin and biotin biosynthetic genes correlating with itaconic acid production is of further interest. Whereas the itaconic acid pathway protein CAD belong to a cofactor-less decarboxylase class [18,19], thiamin and biotin dependent enzymatic steps are present in the glycolysis pathway directly downstream of pyruvate in the direction of the TCA cycle and thus the itaconic acid pathway (*pycA*, *pdcA*: Figure 1). Pyruvate carboxylase (*PycA*) is a thiamin dependent protein and pyruvate decarboxylase (*PcdA*) is a biotin dependent protein [15].

Among the up-regulated group I genes, several plasma membrane genes, including the previously identified MFS transporter gene *mfsA*, were identified which might also function in itaconic acid secretion/detoxification. Moreover, the identification of genes encoding extracellular/membrane proteins and stress related genes may indicate that itaconic acid secretion is related to cell wall modification and acid toxicity. Furthermore, identification of a predicted peroxisomal MFS transporter gene (ATEG_09368) might indicate the importance of additional precursor/product transport during itaconic acid production. The up-regulated mitochondrial copper transporter gene might be related to the positive effect of copper on itaconic acid production as described in Li et al. [5], where copper was found to be positively related with itaconic acid production within a range of 0.005-0.02 mM.

To analyze whether the genes identified based on our clone based transcriptomics analysis were truly specific for itaconic acid production and not for e.g. culture pH, we compared our results with the data from Andersen [12] where the cultivation conditions were quite similar to ours, using *A. niger* as a model, glucose as a substrate and low pH of 2.5 for organic acid production. From this comparison only three genes corresponded to genes identified from our analysis in *A. terreus.* However, even these three genes did not show the same regulation as the ones from our *A. terreus* array, indicating that the genes identified in the *A. terreus* arrays were not selected based on a difference in culture pH. The difference in regulation of the *cipC* gene from *A. terreus* and *A. niger* data sets suggested that the *cipC* expression is not directly pH related. Recently it was shown that *cipC* encodes a stress responding protein in *A. carbonarius*[20], however how this may be related to the expression of the corresponding gene in *A. terreus* under itaconic acid inducing conditions is unclear.

## Conclusion

A systems biology approach was performed to analyze genes relevant for itaconic acid production in *A. terreus* in controlled batch fermentation processes. The transcriptomics analysis identified 33 genes clustered in 4 groups where three groups of genes were positively correlated with itaconic acid production, whereas one group was negatively correlated. In particular the suggested role of PP pathway and glycolysis genes would provide an opportunity to study the itaconic acid production pathway in more detail. Comparing our data with micro-array data from Andersen [12] indicate that the genes identified from our *A. terreus* array were truly itaconic acid specific and not pH specific. In conclusion, the clone based transcriptomics analysis combined with targeted cluster analysis was shown to be an efficient method in uncovering potential functionally related genes.

## Competing interests

The authors declare that they have no competing interests.

## Authors’ contributions

AL carried out the part of the data analysis and drafted the manuscript. MC normalized the raw data of *A. terreus* arrays and helped with the data analysis. PP supervised the study and approved the final manuscript. All authors read and approved the final manuscript.

## Supplementary Material

Additional file 1: Table S1 *A. terreus* itaconic acid biosynthesis related genes clustered by differential expression analysis (Pearson correlation) based on itaconic acid titer and productivity. Click here for file

Additional file 2 **Overview of the fermentations performed under different cultivation conditions as previously described by Li et al. **[11]**.** Click here for file
