# Supplementary material for: A systems biology approach for the identification of target genes for the improvement of itaconic acid production in Aspergillus species
Source: BMC Res Notes. 2013 Dec 4;6:505. doi: 10.1186/1756-0500-6-505 (PMC4235043; doi:10.1186/1756-0500-6-505)
Supplement: Additional file 2 — Overview of the fermentations performed under different cultivation conditions as previously described by Li et al. [11]. [file 1756-0500-6-505-S2.docx]

| Additional file 2- Overview of the fermentations performed under different cultivation conditions as previously described by Li et al. [2] . | | |
| --- | --- | --- |
| **Fermentation** | **Environmental condition^*^** |  |
|  |  |  |
| 1 | Glucose (100g/l) (reference) |  |
| 2 | Fructose as C- source |  |
| 3 | Maltose as C-source |  |
| 4 | Glucose (100g/l) (reference) |  |
| 5 | pH set 3.5 |  |
| 6 | NOT INCLUDED |  |
| 7 | Low glucose (30g/l) |  |
| 8 | O_2_ set point 25% |  |
| 9 | 5* higher Mn |  |
| 10 | Glucose (100g/l) (reference) |  |
| 11 | pH set 4.5 |  |
| 12 | NOT INCLUDED |  |
| ^*^ Fermentations were performed in 5 liter Benchtop Fermentors (BioFlo 3000, New Brunswick Scientific Co., Inc.) at 37 °C. The basic pH regime was initiated at 3.5 and subsequently fixed at 2.3, by 4M KOH (Base) and 1.5 M H_3_ PO_4_ (Acid). In the basic DO (Dissolved oxygen) regime, DO was controlled at 75% on day1, at 50% on day 2, 3, 4 and at 25% on subsequent days (fermentation #1, 4, 10). The following variations were applied to obtain variations in itaconic acid production. In one fermentation culture (#9), 3.5 mg of MnCl_2_*4H_2_O (5 times higher) was used. One of the fermentation (#7) had a reduced glucose level of 30 g/l in the main culture. In two other fermentations fructose (#2) or maltose (#3) were used as the carbon source instead of glucose. In three fermentations #5,6,11, a different pH control was applied. Fermentation #5 and #11 had a pH controled at 3.5 and 4.5 respectively, while fermentation #6 initiated at pH 3.5 after which pH was no longer controlled. In two fermentations #8 and # 12, an alternative DO regime was applied, namely 25% and 10%. Struktol (Schill & Seilacher) was applied as antifoam agent in all cultures throughout the fermentation. | | |
